# Emotion in languaging: languaging as affective, adaptive, and flexible behavior in social interaction

**DOI:** 10.3389/fpsyg.2014.00720

**Published:** 2014-07-16

**Authors:** Thomas W. Jensen

**Affiliations:** Centre for Human Interactivity, Institute of Language and Communication, University of Southern DenmarkSlagelse, Denmark

**Keywords:** first order languaging, second order language, affective stance, ecological naturalization, inter-affectivity, emotion, sense making

## Abstract

This article argues for a view on languaging as inherently affective. Informed by recent ecological tendencies within cognitive science and distributed language studies a distinction between first order languaging (language as whole-body sense making) and second order language (language as system like constraints) is put forward. Contrary to common assumptions within linguistics and communication studies separating language-as-a-system from language use (resulting in separations between language vs. body-language and verbal vs. non-verbal communication etc.) the first/second order distinction sees language as emanating from behavior making it possible to view emotion and affect as integral parts languaging behavior. Likewise, emotion and affect are studied, not as inner mental states, but as processes of organism-environment interactions. Based on video recordings of interaction between (1) children with special needs, and (2) couple in therapy and the therapist patterns of reciprocal influences between interactants are examined. Through analyzes of affective stance and patterns of inter-affectivity it is exemplified how language and emotion should not be seen as separate phenomena combined in language use, but rather as completely intertwined phenomena in languaging behavior constrained by second order patterns.

## Introduction

Emotion and language belong together. Indeed, in this article it will be argued that emotion in fact lies at the heart of language if viewed as an embodied dialogical activity. Still, within mainstream linguistics as well as in communication studies, language and emotion have so far been categorized as belonging to two separate domains that must be kept apart: Language, on the one hand, belongs to the structures of thought comprising an abstract “language system”; it is based on words and representations and it is communicatively deliberate, while emotion, on the other hand, belongs to the body; it is associated with un-intentional reactions, sensations and actions visible in a non-abstract and separate “body language.” This article, however, aims to show the inadequateness, and ultimately false nature, of these dichotomies while pointing to a new way of looking at the relationship between language, emotion, action, and intersubjectivity. It is about time to put an end to unfruitful divorce between language and emotion. They need to be brought back together.

### First order languaging and second order language

New developments in language studies have now made it possible to investigate emotion as an integral part of our language activity rather than studying emotion as a somehow separate phenomenon added to speaking. The recent theoretical developments carving the way for such a proposal have taken place within a variety of new approaches to language, cognition and social interaction such as *distributed language and cognition* (Thibault, 2008, 2011; Kravchenko, 2009; Cowley, 2011a; Rączaszek-Leonardi, 2011; Pedersen, 2012; Steffensen, 2012; Cowley and Vallée-Tourangeau, 2013; Jensen, 2014), *dynamical systems* and *interpersonal coordination* (Bickhard, 2007; Fusaroli et al., 2013b; Fowler, 2014), *dialogism* (Linell, 2005, 2009), *ecological psychology* (Gibson, 1979; Hodges, 2009, 2011), and *embodied and enacted cognition* (Chemero, 2011; De Jaegher and Di Paolo, 2007; Anderson et al., 2012; Di Paolo et al., 2013).

The key notion in the present work is the term *languaging*. It originally stems from the early works of Maturana (1970) and has recently been revived and redeveloped by a number of scholars working within the distributed language group (Love, 2004; Linell, 2009; Cowley, 2011a; Pedersen, 2012; Steffensen, 2012). In particular the term has been elaborated in various works by Thibault (2005, 2008, 2011), and it is this particular version of the notion of languaging that will be adopted in this article^1^. In his 2011 article Thibault argues that the recent developments within distributed language studies represent:

..a renewed attempt to better understand the materially embodied, culturally/ecologically embedded, naturalistically grounded, affect-based, dialogically coordinated, and socially enacted nature of languaging as a form of whole-body behavior or whole-body sense making (p. 211).

This view attempts to capture the activity bound character of language as its primordial feature. Languaging involves a complex coordination of multiple activities emphasizing the dynamics of real-time behavioral events that are co-constructed by co-acting agents. For that reason languaging—language as an activity—is promoted as a *first-order* phenomenon, whereas what is usually referred to as language within linguistics—language as a symbolic and rule-governed system—is seen as a *second-order* construct or constraint on languaging behavior. The term “language” therefore becomes an umbrella term encompassing both first and second order as two different but intimately related dimensions in this specific kind of behavior.

Importantly, this approach entails an *inversion* of the traditional ontological order of language saying that firstly we have a “language system” which is then turned into use by “language users.” This is rejected arguing that first of all there is *activity*, and out of this languaging activity “grows,” on longer evolutionary as well as socio-cultural timescales, language as a symbolic system-like constraint that highly influences languaging behavior. This shift is crucial because it re-conceptualizes our general understanding of “language.” Traditionally, within folk understandings as well as within linguistics, we look upon and comprehend language as a combination of system and use (with the system as the primary ontological phenomenon and the use as an epi-phenomenon). From a distributed perspective however, we can see language as an activity system; that is comprised of first order activity and second order constraint. i.e., “we depend on dynamics first and symbols afterwards” (Cowley, 2011a, p. 11). In that sense the term “language use” implies a pre-established system whereas *languaging* designates activity or behavior as the primary ontological feature of language while also acknowledging the socio-cultural constraints making this activity something distinct—or different from other types of activity or behavior.

This article is chiefly an examination of the affective and emotional dimension of languaging dynamics of face-to-face interaction (i.e., speaking, hearing, gazing, gesturing, mimicry, postural sway, and so forth) while also considering how these types of activity are constrained by second order patterns^2^. The theoretical claims put forward in this work are developed on the basis of thorough analyses of empirical data consisting of video recordings of different situations and subsequent transcription that allow for detailed investigation of the inter-bodily dynamics of human dialog.

### Emotion as part of languaging

Within the growing literature on distributed language and cognition (Thibault, 2008, 2011; Kravchenko, 2009; Cowley, 2011a; Rączaszek-Leonardi, 2011; Pedersen, 2012; Steffensen, 2012) the close relationship between emotion and languaging has often been implied (e.g., languaging as “affect-based” in the Thibault quote above). Still, a more thorough attempt to investigate the intricate connections between emotion and languaging remains to be seen. This article is a first step in this direction by relating and specifying the languaging approach in terms of emotions in social interaction. It will be argued that emotion is not separated from language—as an independent non-verbal component to verbal communication as it is often laid out—nor can emotion be regarded as merely a secondary function of language. Instead *emotion and affect are integral parts of languaging behavior*^3^, or rather languaging is whole body activity *including* emotion.

On a fundamental level we feel in conjuncture to the movements of ourselves as well as other people: We see, hear and experience other people's emotions in and through their whole-body movements (facial, gestural, postural, and vocal) and likewise we enact emotions by altering our voices, moving our bodies, using our facial muscles, making gestures, or touching each other (Colombetti, 2014). Thus, emotions and emotional experiences are inherently tied to bodily sensations. Indeed it is virtually impossible to imagine an emotion without a bodily sensation as famously argued in relation to fear by William James:

What kind of emotion of fear would be left, if the feelings neither of quickened heart-beats nor of shallow berating, neither of trembling lips nor weakened limbs, neither of goose-flesh nor of visceral stirrings, were present, is quite impossible to think (James, 1884, pp. 193–194).

Furthermore, a fundamental quality of emotions is their “ability” to ascribe value to experiences. Through emotions we experience something as “something”—fearful, exciting, boring, scary, attractive, or repulsive. As several neuro-scientific studies of people with brain damage have shown, without emotion the world appears “gray” and uniform with no appeal to act upon it (Damasio, 1996). Within such studies emotions are examined in relation to the human brain “as complex collections of chemical neural responses forming a distinctive pattern” (Damasio, 2003, p. 53). In short emotions can be seen as complex neural, chemical, and behavioral patterns functioning as feedbacks on encounters or situations processes by which our bodies assess their state and make adjustments to maintain their homeostasis. Thus, in this sense, which is the position taken in this article, emotions *are* in fact movements; not just within us however, but also movements that connect experiences with situational affordances:

Emotions are processes of organism-environment interactions. They involve perceptions and assessments of situations in the connected process of transforming those situations. The body states connected with feelings are states of both response and remaking of experience. I say, “I'm fearful,” but this really means “The *situation* is fearful”; fearfulness might appropriately be described as an objective aspect of the situation *for me at this moment*. (..) In short, emotions are both *in us* and *in the world* at the same time. They are, in fact, one of the most pervasive ways that we are continually in touch with our environment (Johnson, 2007, p. 66).

However, in order to relate these processes directly to language a re-specification of our conception of language is called for. And this is what the notion of languaging offers: As part of our languaging behavior, parts of our whole-body sense making, emotions are enacted as evaluative processes, intersubjective positions, and possibilities for action^4^. In that sense emotions are part of a human-environment system. They are part of our ecology as properties of whole situations, including individuals and environmental structures. To sum up, given that emotions are seen, not as individual inner states, but as processes of organism-environment interactions, and given that languaging is seen, not as an abstract semiotic system, but as dynamic adaptive behavior, emotion is to be seen as an intrinsic part of languaging itself. Indeed, it is impossible to fully understand languaging as behavior without considering emotion.

### Structure of the article

Overall the article can be divided into five major parts: Following this introduction, there is a critical examination of the way emotion has been addressed by separating it from language within linguistics and communication studies (section Traditional Obstacles in Integrating Language and Emotion). This is followed by a more elaborate treatment of a combined dialogical/ecological approach to language and cognition with a specific focus on an how emotion can be seen as part of languaging (section Languaging). Section Analyses is the empirical part, consisting of analyses of video recordings of real life social interactions investigating the claims put forward in the previous section. Finally, in section 5 the analytical findings and theoretical claims will be put in perspective in relation to the study of emotion and cognition and the methodological challenges of this new approach will be discussed.

## Traditional obstacles in integrating language and emotion

Why is it then that the phenomenon we call language is commonly understood as something separate from emotion? Or rather, what is it in our understanding of the notion of “language” that makes it separate from that of emotion? An attempt to answer these huge questions, while staying within the space limitations of this article, has to operate with a strict focus. Let us therefore limit our focus to four widespread views on language and communication that indirectly have come to function as obstacles for a more integrated view on emotion and language: (1) A view on language as a *code-like system*. (2) A conception of language as a phenomenon first and foremost based on *words* resulting in distinctions between *language* vs. *body language*, and *verbal* vs. *nonverbal* communication. (3) A view on language and communication as a *transfer of information* from a sender to a receiver. (4) A view on language as a *social phenomenon* through and through that can be treated without any consideration of its biological dimensions.

Let us now take a closer look at these obstacles.

### Obstacle 1: language as a code-like system

Twentieth century linguistics was dominated by powerful form-based theories of abstractions like structuralism and generative grammar that ended up excluding the dynamics of real time language behavior as a relevant study of object (Harris, 1987; Linell, 2009). As it has often been noted in the history of linguistics (Lyons, 1981) the two major components in Saussurean linguistics: *langue* and *parole*, share many similarities with the Chomskyan notions of *competence* and *performance*, in the sense that the proper object of study became “language” as a hidden set of structured forms underlying the various kinds of language use. The language system is conceived as either an autonomous system (langue) or a specific module in the brain (competence). In both cases the key is that the *language faculty* is separate and must be studied in its own right apart from the messy dialectics of real-time speech production and comprehension. As a consequence the focus on an idealized system of linguistic knowledge left no room for the role of emotion or affect; emotion was categorized as a phenomenon that *by definition* is excluded from (the study of) language.

Looking back, these abstract theories of language have been heavily criticized for losing sight of the way language is actually used and for completely neglecting the role of the context (Levinson, 1983; Chafe, 1994). As a consequence, since their heyday a wide variety of usage based approaches to language have appeared. There is, however, still a massively prevalent tendency to think of language in terms of system and use respectively^5^; the premise being that if studying language you can choose to focus on one or the other, but the fundamental division in system and use is—almost—unquestionable. The problem however in accepting this division, even for usage based theories, is that the underlying assumption is that the system is the foundation (or the essence) while the use is a changeable epiphenomenon. The theoretical consequence is that emotion can never be part of language itself; it can only be added as an extra non-linguistic device in language use.

### Obstacle 2: language as first and foremost based on words

In his, 2005 book Per Linell describes a *written language bias* concerning a strong tendency in linguistics to describe and understand spoken language in the terms of written language—resulting in a fatal lack of awareness of the distinct characteristics of spoken language. It has resulted in the common assumptions that writing and speaking are only different external manifestations of the same underlying “language” (langue, competence, conceptual system, etc.) and thus that writing and speaking basically share the same task of expressing human thought—albeit in different ways. A further consequence has been a *reification* of language. Language is seen as a phenomenon that by definition is based on words (or other lexical items), and subsequently sentences and grammar, as in written language^6^. That is, words or other lexical items, function as designators of fixed and well-defined meanings (except when deployed in metaphorical or indirect ways). Words are treated as separate entities that function as representations of meaning. As a consequence there is a separation between what is intrinsic to the meaning of words and what is somehow seen as being outside this confined linguistic meaning.

This view lives on in the popular and widespread (common sense) distinctions between *language* vs. *body language* or *verbal* vs. *non-verbal* communication. The former are based on words, the latter on something else (bodily practices) than words. Body language or non-verbal communication is by definition something separate from language concerning unintentional sensations or feelings that contain an “unspoken meaning”^7^. Whereas body language is exclusively defined as *behavior*, not language (Boyes, 2005), the concept *paralanguage* is defined as meta-communication more directly related to language (Poyatos, 1993; Van Berkum et al., 2008). Still, it relies on a distinction of the linguistic content in itself (*what is said*) as separate from the variety of ways, typically involving prosody, pitch, volume, intonation etc., in which something is said or communicated (*how it is said*) (Thibault, 2008).

The theoretical consequence is again that the numerous, and affective laden, ways in which words are deployed (negotiated, interpreted, explored enriched, etc.) in the meaning dynamics of actual talk becomes detached from “language itself.” Therefore, emotion and affect is treated as something that can only modify, emphasize or nuance meaning by its virtue of not being language.

### Obstacle 3: communication as transfer of information

The classical idea within communication studies is still that communication can be captured as a transfer of information between individuals (Weaver and Shannon, 1963). This notion rests on the idea that *something* is communicated and furthermore that this “content” is of a somewhat stable character. This idea has been analyzed in terms of *the conduit metaphor* in which language is viewed as a “conduit” conveying mental content between people (Reddy, 1979). It is metaphorically construed as if, whenever people communicate, they “insert” their mental contents (meanings, thoughts, concepts, etc.) into “containers” (words, phrases, sentences, etc.) whose contents are then “extracted” by listeners. Again it is worth noticing that this conceptualization rests on the highly problematic notion that meanings of utterances as somehow internal and distinct from their unfolding or deployment^8^.

Interestingly there is a strong parallel to the way emotions, or rather emotional expressions and emotional communication, have been studied. The most obvious example is the way in which the human face has often been described as a sort of “mirror” of our emotional states. Thus, facial expressions are widely considered the most reliable source for studying emotions dating all the way back to Charles Darwin's seminal work *The Expression of the Emotions in Man and Animals* (1998/1872). More recently the psychologist Paul Ekman has conducted several studies on the alleged universal correspondence between basic emotions and specific facial expressions (Ekman, 2006, 2007)^9^. However, this type of research in facial expressions rests heavily on a Cartesian division between the inner emotional state and the outer emotional expression: Emotions are hidden inside us and sometimes our facial expressions reveal this “inner landscape.” Thereby the expressive or communicative part becomes only an outer byproduct of the inner source—the emotions themselves. Furthermore, there is a tendency to view emotions as revealing as well as “real.” They can be trusted (unlike language) exactly because they are “involuntary not intentional” (Ekman, afterword in Darwin, 1998/1872, p. 372)^10^. They disclose our inner motives and desires and thereby send an unintentional “message”: “We don't make an emotional expression to send a deliberate message, although a message is received” (ibid: 373).

For this reason this approach has also been criticized and re-thought within communication studies:

The fact that people can and do alter the expressions of even the primary emotions suggest that emotion display or *emotion expression* may be more aptly termed *emotional communication*, in the sense that emotional information, like other types of information, is shaped for audiences. (..) Emotions may (or may not be) be activated internal states, but when they are communicated, they are packaged in ways that are consistent with other communication practices (Metts and Planalp, 2003, pp. 348–49).

Still, even though the authors attempt to free themselves from the dualistic tension in the term “emotional expression” they get caught up in the communication transfer model. Emotional communication is still understood and conceptualized in terms of a sender and a receiver. Indeed, the whole argument governing the division of emotional communication into different “channels” or “cues” (physiological, bodily, vocal, and facial cues) is flawed by its own terminology. Thus, the sheer notion of emotional cues still entails a view on emotions as an encapsulated entity originating within the individual and then being brought into public light through different devices. Emotions are described as “information” which is then “shaped for audiences” when being communicated—exactly like the linguistic meaning is described within communication models. To sum up, the notion of emotional communication is only possible by means of dualistic separations of “inner emotional states” from the outer social communication of those states, and likewise a separation of the specific “emotional cues” (body language) from the “real language.”

### Obstacle 4: language as a purely social phenomenon

This last obstacle reflects a tendency which is present in varying degrees within different contemporary language studies, such as *linguistic anthropology* (Wilce, 2009) *discourse analysis*, *discursive psychology* (Potter, 1996) and the so-called *third wave sociolinguistics* (Eckert and Rickford, 2002), to postulate that most, if not all, aspects of reality are constituted, embedded, and maintained in and through language. What we call “reality” is socially negotiated and linguistically constituted which means that we do not have access to any kind of reality outside of our linguistically determined experience. This view rests on the assumption that language does not represent a given reality “out there” but rather constitutes our experience of reality.

The basic idea that language is first and foremost a practice and cultural resource which gains its meaning, not from representing thoughts or ideas, but from what it does in contextually defined situations, actually does have many points in common with a distributed “languaging approach.” Still, this purely social, or constructionist, view often comes with an unfortunate tendency to reject natural or biological phenomena as having a meaning outside of conceptual treatments. Put a bit crudely, it implies that language defines the scope of our experience and therefore we only have access to “natural” phenomena in and through our language use. Or rather, they only gain meaning by being conceptualized through language. This creates a focus on *language ideologies* (Bauman and Briggs, 2003), among them how emotions are conceptualized in our language use. Despite the relevance and interesting findings of such studies there is a tendency to reduce emotion to a matter of words or ways of talking:

Discursive psychology, for example, examines emotion vocabularies and refers to emotion discourse as a “way of talking.” “Instead of asking the question, ‘What is anger?’,” Harré writes, “we would do well by asking, How is the word ‘anger’ actually used in this or that cultural milieu and type of episode?” (Maynard and Fresse, 2012, p. 93)

The premise of such studies lies in the constructionist assumption that our access to emotion is mediated and constituted by our language use. Emotions are only “emotions” when called “anger,” “joy,” “embarrassment,” and so forth. Thus, emotions become intellectualized as a matter of words and concepts and the result is that there is no independent (emotional) reality outside of language. Instead of widening, or redefining, the notion of language, as inherent in the notion of languaging, language becomes detached from its embodied characteristics and emotion is locked in the confined room of emotion words. Likewise, bodily actions and movements are in many constructionist analyses (Harré, 1986; Gergen, 2009) treated as first and foremost a by-product of verbal discourse and social conventions which, in the end, results in a *social reductionism* that leaves the embodied biological dimensions of emotions fundamentally unexplained.

Now, from the vantage point of this article it is vital to avoid all of these obstacles separating emotion from language and instead strive toward an ecological naturalization that sees language “as fully integrated with human existence” (Cowley, 2011a, blurb), implying, among other things, that emotion and affect can be embraced as integral parts of languaging behavior. Let us now have a closer look at such an approach.

## Languaging

### An ecological naturalization

First of all it is important to clarify that an ecological naturalization (Steffensen and Cowley, 2010; Thibault, 2011; Steffensen, 2014) is by no means an attempt to reduce culture, sociality and language to biology, neurology or physics as implied in some previous attempts on naturalization (Pinker, 2003). On the contrary an ecological naturalization goes against any sharp distinction between the socio-cultural and the natural sphere. In relation to the present work, the key ambition is to present a study of how emotions can be analyzed in situ without committing to *either* a biological or a social standpoint that respectively excludes the other. Instead, inherent in the notion of languaging proposed here is the tenet that language, at the same time, is a cultural organization of processes and naturalistically grounded in human biology implying that:

..there is no inherent contradiction between seeing language as biogenic and as social, simply because sociality is our human way of being nature. This assumption both precludes the bio-reductionism that ignores supra-individual (i.e., social or cultural) dynamics and the socio-reductionism that ignores the metabolic and ecological foundations of human existence (Steffensen, 2014).

Secondly, this ecological viewpoint crucially affects the re-thinking of the notion of language, conceptualized as first order dynamics and second order patterns, as mentioned in the introduction. Real time adaptive flexible behavior and coordinated activity is referred to as first-order languaging (putting weight on the fact that language arises from activity); this activity however presents itself (on a phenomenological level) as words and utterances with meanings and connotations and so forth, i.e., as second order language. Contrary to a representational view on language however, it is crucial to bear in mind that “speaking does not refer to the world; it *causes* an experience that happens to coincide or not with the narrow situation or the larger reality such as it is enacted” (Bottineau, 2010, p. 278). Thus, the meaningful patterns and configurations of speaking arise because we, as bio-social beings enmeshed in specific social realities, are accustomed to take, what Stephen Cowley has coined, a *language stance* (Cowley, 2011b). We learn to scrutinize and discriminate between different sounds (and movements) so that we hear vocalizations as words in the process of being enrolled in an ecological reality. In a complex bio-social environment, bodies, physical artifacts, words, embodied movements (gestures, gazes, mimicry, postural sway, etc.), social norms, and other sociocultural resources all function as enabling conditions or *affordances* (Gibson, 1979; Hodges, 2009) for human action. Thus, put a bit crudely, the focus shifts from abstract forms (as in traditional linguistics) to a reconsideration of how “we perceive bodily events as wordings. Emphasis on coordination allows due weight to be given to the fact that languaging predates literacy by tens-of-thousands of years. By hypothesis, all linguistic skills derive from face-to-face activity or languaging” (Neumann and Cowley, 2013, p. 18).

This ecological approach does not need to mark a sharp line or discrimination between (what is usually called) a natural or social/cultural reality. Instead the distinctions or dualisms between the biological vs. the social and the here-and-now vs. the grand scale formations are challenged by grounding languaging in bodily co-experience while at the same time being sensitive to overreaching cultural and social constraints on language.

### Languaging, primary intersubjectivity, and language

As laid out by Paul Thibault the recent movements within distributed language studies positions languaging as intimately related to intersubjectivity and affective attunement:

“Human language is seen more and more as a suite of flexible and adaptive behaviors that are based upon a naturalistically grounded intersubjective sensitivity to the bodily dynamics (movement) of others and the sensorimotor coupling relations between persons and their worlds that result from this in the intersubjective matrix” (Thibault, 2011, p. 212).

In the same vein, in a recent publication within embodied and social cognition, Joel Kruger refers to an older study of breastfeeding (Kaye, 1982): “the infant's earliest and most complex form of social interaction. The rhythmic cycles and back-and-forth interplay of breastfeeding appears to play an important role in the infant's social cognitive development… Within the dynamics of this exchange, mothers sculpt the infant's attention: their behavior is organized by the mother's touch and physical prompting. The infant is guided to notice salient environmental affordances by the jiggling (e.g., the nipple affording feeding) that, in light of her underdeveloped endogenous attention and lack of behavioral organization she might not otherwise pick up” (Krueger, 2013, p. 43).

It seems obvious that the contours of languaging, in its most basic form, are definitely grounded in such early intersubjective behaviors. Of course, later in the course of life it expands and gains an enormous complexity by being enmeshed in the socio-cultural reality, as described in the previous section. Thus, what is referred to in the present work as “languaging” overlaps, to some extent, with what other scholars, primarily concerned with bodily behaviors only (Gallagher, 2005; De Jaegher and Di Paolo, 2007; Gallagher and Zahavi, 2008), call *primary intersubjectivity* (Trevarthen, 1979). However, in this work “languaging” is put forward since the specific research interest and focus is different. It is a focus on showing the continuity between bodily engagements and activities including speaking and verbal behaviors—and thus second order. That is, bodily activity in the here-and-now which is always already being constrained by situation transcendent elements emanating from larger socio-cultural timescales. The commonly learned second-order language shows up in the flow of first-order languaging, shaping and constraining the possibilities for sense-making therein, though not exhaustively determining or explaining them. In that sense languaging behavior is infused with second order patterns; thus the first/second order distinction is not a clear cut separation like the traditional distinction between system and use.

Furthermore, there is a tendency within both primary intersubjectivity approaches (Trevarthen, 1979; Gallagher and Zahavi, 2008; Krueger, 2013) as well as embodied and extended approaches to cognition (Clark, 2008; Chemero, 2011) to *underthematize language*, and thereby not attempt to explain how language more specifically relates to our bodily engagements. Many scholars who seem quite progressive in relation to cognition, perception, emotion etc. still maintain a somewhat traditional view on language as “a tool for thinking” (in traditional views) or (in more modern versions, see Clark, 2008) a way of extending our minds into the world, and thereby neglecting the activity bound character of language (see Steffensen, 2009; Fusaroli et al., 2013a; for a similar critique). Whereas the languaging approach allow us to see language as first and foremost an activity; it “is a doing” (Cuffari, 2014, present volume) intimately tied to affective attunement while also being constrained by second order patterns.

### Affective stance and inter-affectivity

In opposition to traditional dualistic conceptions of emotion as “inner states” and behavior as “outer conduct” there is a long and rich phenomenological tradition of dealing with perception, action and emotion as intertwined phenomena by, among others, Maurice Merlau-Ponty:

I do not see anger as a psychic fact hidden behind the gesture (..) The gesture does not make me think of anger, it is anger itself. I perceive the grief or the anger of the other in his conduct, in the face or his hands, without recourse to any inner experience (Merleau-Ponty, 1964/1992, pp. 48–49).

The important point, made already more than half a century ago, is that we do not, as commonly thought, infer inner emotional states on the basis of (an interpretation of) outer behavior; rather we perceive emotions directly in our interlocutors. Emotions come about as behavioral patterns, or put another way, they are in the behavior, not a product of or something to be drawn out of the behavior. Relating this to languaging and human interaction and emotion we can say that, in interaction, we perceive emotions directly in order to do things. Gestures, facial displays, posture, wordings, or simply whole-body languaging acts, generate affordances for trajectories of further action in human dialog (Hodges, 2009). Interaction is constantly pushed forward by actions that invite or afford further actions by; here emotions play a crucial role as the “grease” keeping these dynamics going. In that sense human dialog is often, in varying degrees, infused with, what Karl Bühler called “communicative valence” (*kommunikative Valens*—Bühler, 1934, p. 31. Taken from Caffi and Janney, 1994):

During interaction, we tend to perceive others as “opening up” or “closing down,” being responsive or reticent, making signs of approach or withdrawal; we perceive their relative strength or weakness, their fuller or lesser presence, their attentiveness or disinterest. All such perceptions are rooted in, and depend on, emotive displays. (..) It is the capacity, for example, to view “positive” behavior as a possible starting point for agreement or cooperativeness, “negative” behavior as a possible starting point for disagreement or conflict. (..) In all cases, the interpretation of emotive activities involves an appreciation of interpersonal relations and self-presentation (Caffi and Janney, 1994, p. 329).

This aspect of human interaction is often described in terms of stance taking (Du Bois, 2007; Goodwin, 2007; Goodwin et al., 2012). According to John Du Bois, when we express opinions, and/or display affect, three dimensions are at stake simultaneously; *evaluating* the topic we are talking about, *positioning* ourselves with respect to topic and others, and *aligning* or *dis-aligning* with our interlocutors. There is, however, a quite narrow focus on words and a somewhat individualistic point of view in (parts of) the stance literature^11^; consider for instance these lines from Du Bois' *The stance triangle*: “One of the most important things we do with words is take stance(..) Stance can be approached as a linguistically articulated form of social action” (Du Bois, 2007, p. 139). From the vantage point of this article it is crucial to widen the scope of stance so as to investigate affective stance as part of (whole-body) languaging behavior and intertwined with the dynamics of human-environment-systems. Stance is the perfect example of languaging as whole-body sense making; processes of evaluating, positioning and/or aligning/dis-aligning are by no means restricted to “the use of words” (even though they often play a part) but involve whole bodies engaging in adaptive flexible behavior.

Affective stance is crucial in understanding languaging as attunement to the environment in and through coordination of behavior (Bickhard, 2007; Fusaroli et al., 2013b). Languaging is about coordinating dynamics; it is “something we *do together*” (Fusaroli et al., 2013a, p. 2). Taking this perspective a step further, in a recent article on gesture in interaction Böhme and colleagues investigate how we do affective coordination together, coined as *inter-affectivity*.

Affect in face-to-face communication is assumed to manifest itself as embodied inter-affectivity. Our analyses will document that affect is in fact a dynamic and shared “in-between” phenomenon, jointly created by the participating interlocutors. Therefore, an *interactive expressive movement unit* is a sequentially organized product of joint gestural activities of co-participants in an interaction, which, by definition, entails more than one gesture unit (Böhme et al., 2014, p. 2116—italics in original).

This notion of inter-affectivity, challenging the idea of affect and emotion as properties of individuals, in turn makes it possible to question the traditional clear cut distinctions between Self and Other as two separate entities that can only communicate be means of “emotional cues” or “channels.” Rather, human interaction can be seen as an unfolding of a “temporarily coordinated functional whole, consisting of two sub-systems (Rączaszek-Leonardi, 2011). A consequence of this is that the unit of analysis shifts from the interpretation of individual doings and the causal link between separate actions to a more *systemic view* considering human interaction as a *dialogical system* (Steffensen, 2012) which can be seen as “systems of co-present human beings engaged in interactivity that bring forth situated behavioral coordination (or a communicative, structural coupling) (Steffensen, 2012, p. 513). Such behavioral coordination is infused with affective valence and emotion from the very outset. Adaptive flexible behavior is all about adjusting, attuning, directing, opposing or contrasting behavior within a human-environment-system, or human-human-environment-system. Or put in another way, emotions can be seen as the glue of dialogical systems.

## Analyses

### Method and transcription

Central to the notion of languaging, as previously described, is the inclusion of embodied actions of all sorts: posture, gaze, gesture, facial movements, voice quality, in- and out-breaths, etc., are all important parts of first order languaging. This of course needs to be reflected in the methodological praxis in general, and specifically for this work, in the transcribing and notation of interactional data.

However, there can be no such thing as all-encompassing transcription; for instance the notation of facial movements and gesture in the present work is by no means as detailed as studies focusing solely on these phenomena, for example by using close ups on each participants face and hands. In this case, only one camera for each recording was used. Still, as mentioned earlier the primary research questions for this work concern languaging behavior in its totality, not the specific role of facial movements or gesture as such. A basic model of the transcription system developed by the conversation analyst Jefferson (2004) is employed here which include notations of basic prosodic features, such as pitch, volume, speed, intonation, and tone of voice (i.e., smiley or crying voice). In many conversation-analytic studies the verbal and vocal activities are supplemented with comment lines of descriptions of embodied activities. Still, a serious challenge for developing a specific methodology for analyzing languaging in situ is the traditional outset in words and individual talking turns inherent in both the notion of speech acts as well as (to some degree) in conversation analysis (Searle, 1969; Hutchby and Woofit, 1998). As noted by, amongst others, Per Linell and Sarah Bro Pedersen a word and line based transcription (with bodily movements only appearing as comments) can in itself be seen as proof of a written language bias (Linell, 2005; Pedersen, 2012). Furthermore, to some extent this procedure (involuntary) reflects a tradition in linguistics that endows words and verbal behavior with a certain privileged status. Nevertheless, since we cannot go back in time and be present in the flow of interaction as it took place, we need to be able to capture and represent what went on. For the sake of recognizability this often means reading a word based transcription perhaps combined with notations of bodily movements.

Another way to go about it however is to combine words based transcriptions with images. Images have the advantage of favoring an in situ impression of the interaction instead of a retrospective description; they show the dynamics instead of trying to explain them. For these reasons the verbal transcriptions are combined with images paving the way for an analysis of these conversations as instances of whole-body languaging behavior. The verbal utterances are presented in the Danish original first and then translated into English in the following line (in italics). A complete overview of the transcription symbols is attached as an appendix to the article. Still, it needs to be said that there is a tension between the notion of languaging as whole-body sense making and this CA inspired model of transcription that is in need for clarification and further development in future works^12^.

### Analysis: affective stance in languaging

The following example is taken from a larger recording from a Danish school for children with special needs^13^. M and E, a pair of twins diagnosed with intellectual disabilities, and a speech and language therapist are sitting around a table playing a card game. It is a board game with different cards depicting various objects, animals and social situations and the objective is to train the verbal skills and social knowledge of the children. Leading up to the sequence below M has drawn a card and is now supposed to say what it depicts.

**Figure:**
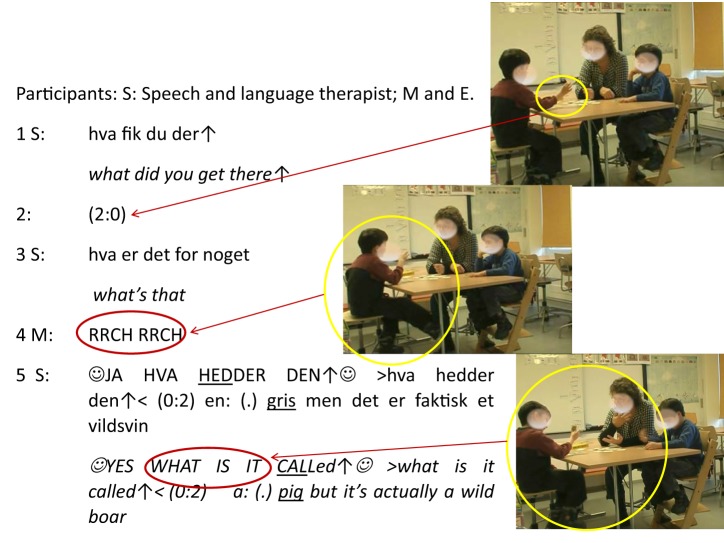


In the middle of the sequence something unexpected happens: instead of delivering a verbal answer to the two questions posed by S (in line 1 and 3) M suddenly performs a variety of (bodily) languaging actions (see second picture). Up till that point M has been sitting still while holding out the card with his right hand for both him and the other participants to see (see first picture). But all of a sudden the intensity changes in the inter-bodily dynamics between M and S. A series of affective movements start unfolding beginning in line 4 with M becoming highly energetic: throwing his torso back and forth, kicking under the table, smiling and moving his head while at the same time with high volume uttering two distinct sounds (RRCH RRCH) resembling the sound of pigs. Immediately the activity level of S changes as well. In the first half of line 5 her eyes widen significantly while gazing directly at M; she smiles and starts speaking with a distinct smiley voice with high volume, emphasis and rising intonation (see third picture). Together these rapidly evolving and tightly coordinated inter-bodily dynamics of M and S build an affective alignment. An alignment that emerges from the totality of the inter-actions, not just as a result of separate individual actions, but as an overall pattern or configuration of expressive movements (Böhme et al., 2014), vocal sounds and wordings that emerges as shared *inter-affective experiences* of intense involvement, joy, and excitement. As depicted by the yellow circle in the last picture both M and S are complete engaged in their inter-affective movement dynamics (gesturing, moving their upper bodies, smiling, grimacing, and gazing at each other) that, taken together, build a shared affective encapsulated by the yellow circle. Thus, the yellow circles means to depict the affective development from M's (individual) gesture and whole-body movements to the inter-affective coupling between S and M in the last picture^14^.

Furthermore, it is crucial to pay attention to the sequential placement of M's initial languaging actions. They are embedded in the ongoing structure of the interaction and performed in line 4 at exactly the point in which a traditional verbal answer would be expected. But instead of stating verbally what is on the card M is *acting* the depicted content by uttering pig-like sounds, kicking under the table and throwing his torso back and forth. It can be seen as a whole-body languaging act of *showing* instead of *telling*. Indeed, these whole-body movements are an instance of *affective stance taking* embedded in the immediate environment and arising from ongoing processes of interaction. As described previously, stance is traditionally understood and described within the framework of words. In this case however, by letting whole-body actions replace wordings M takes a stance that immediately affords an alignment by S. In acting the answer instead of just saying it M indirectly *evaluates* the object as well, i.e., the predefined task at hand and the way the answer is meant to be delivered. Thus, this whole-body languaging behavior redefines the rules in a creative way and thereby *positions* M in relation to the game activities, which in turn enacts an inter-affective space between M and S that *aligns* their stance taking and enhances an immediate intersubjective understanding between them.

#### First order languaging constrained by second order language

Focusing on the second half of the response of S however, reveals the short lived character of this intersubjective alignment: S's confirming response in the beginning of line 5 is quickly repeated only this time without any of the initial prosodic features such as smiley voice, high volume and rising intonation: ☺YES WHAT IS IT CALLed↑ ☺(.) >what is it called↑<), i.e., this repetition works more as a more straightforward request for a verbal answer. In other words, the first-order whole body stance taking is quickly constrained by a verbalized (second order) request. The here-and-now languaging behavior becomes enmeshed in the prerogative—or the second order constraint—of the socio-cultural function of the game: To train the verbal skills of the children. The initial acknowledgement of S had a function: it cooperated in establishing an intersubjective alignment. Then, there were renewed possibilities; room for trying things that are hard and difficult, namely verbal depiction, which is the aim of the game and possible as S is willing to redefine the rules to achieve the goal. For a brief moment S had acknowledged that whole-body languaging is indeed language, meaningful and even powerful. At the same time however, verbal language is needed in this social learning activity, as well as in society in general, to accomplish certain tasks.

In relation to this example the consequence is that an embodied emotional languaging response needs to be enrolled in second order norms and patterns in order to gain recognition and acknowledgement, i.e., it needs to be verbalized. Thus, in the last part of line 5, after a mini pause of 0.2 s, S provides this requested verbal answer herself. She “takes a language stance” and thereby transforms the bodily actions of M into a recognizable verbal pattern naming and categorizing the action of M as depicting “*a: (.) pig but it's actually a wild boar*.”

#### Summary

This example explicated how:

- An increase in the intensity of inter-bodily dynamics formed a space of inter-affectivity, within which whole-body languaging actions replaced a verbal answer functioning as interactional affective stance taking.
- The affective stance taking involved evaluation, positioning and alignment even without the use of words. The properties of stance, normally investigated in verbal language, functioned as an integral part of this languaging behavior.
- Likewise the affective dimension was from the beginning built into these languaging actions, not added to them as an extra nonverbal component. Thus, what is often described as “para-linguistic aspects” such as prosody, facial or upper body movements, are to be seen as part and parcel of first order languaging.
- The whole body affective languaging behavior was constrained by second order language and norms in the responses of the speech and language therapist pointing to socio-cultural function of the game.

In the next example we will investigate further how affect and emotion are built into languaging behavior in the phenomenon of laughing while also being constrained by second order.

### Analysis: the ecology of laughter

Laughter in interaction is an intriguing phenomenon in relation to emotion and affect. It is tempting, and therefore common, to consider laughter as a spontaneous and individual phenomenon; a force of nature that sometimes get the better of us resulting in individual single outbursts of laughter. On the other hand, laughter is commonly experienced as contagious. It rapidly spreads among interlocutors^15^, and in this regard it can be seen as a shared phenomenon that evolves in the intersubjective space between people. Furthermore, in a number of studies the conversation analyst Gail Jefferson has shown how laughter in interaction can be regarded as an activity that invites participation: *“speaker himself indicates that laughter is appropriate, by himself laughing, and recipient thereupon laughs”* (Jefferson, 1979, p. 80—italics in original). Thus, an interlocutor invites others to participate by the act of laughing itself, and furthermore, if the interlocutor does not join the laughing, or only laughs momentarily, the laughter of first-speaker lasts significantly shorter (Jefferson, 1984). In this sense, laughing in interaction is by definition something we do together, and for that reason solo-laughter is not common, nor acceptable, for too long in social interaction. This can remind us that there is much more to laughing than spontaneous and individual outbursts; on a fundamental level laughing is grounded in an ecology of inter-affectivity. It is integrated into the languaging behavior and profoundly tied to the bio-social interworld (Linell, 2009) of perceptions, bodily actions and social attitudes of interlocutors and embedded in interactional structures.

This longer sequence comes from a larger set of recordings of couple's therapy sessions featuring a therapist and married couples^16^. As an introductory exercise this couple is asked to mention one thing about the other that they appreciate and value. This request however, is followed by a considerable pause of 3 s in line 1, which is subsequently broken by the starting laughter-and-talking. Apparently, the silence following what perhaps ought to be an easy task for a married couple creates a contrast that provokes laughter even though it also might appear as problematic^17^:

**Figure:**
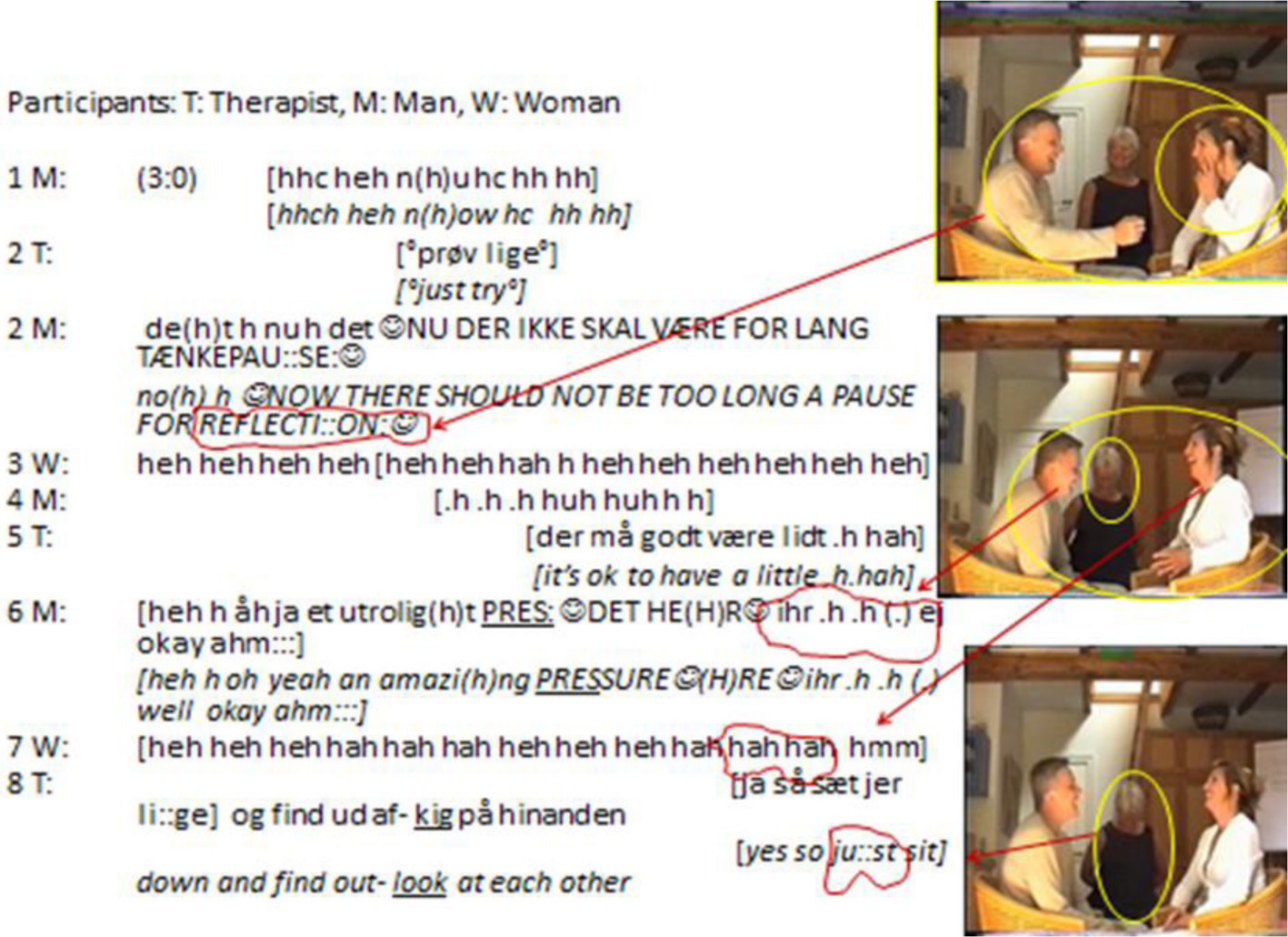


#### Laughing as a gestalt of shared expressive experience

In this sequence the laughing emerges gradually from initial outbreaths and “laugh particles” interpolated within wordings in line 1 over the increase in volume, stress and smiley voice in line 2 to the eruption and flow of a full-fledged laughter in line 3– 7 (see second picture) until it suddenly stops in the overlaps of line 7 and 8 (see third picture). It lasts almost 8 s and has a clear trajectory. The distinct in- and outbreaths evolve in a rhythmical pattern that is completely intertwined with the inter-bodily dynamics of speaking, tone of voice, gesturing, postural sway, facial displays, gazing at each other or into the room, closing one's eyes and even tactility (gathering hands and touching one's face).

In line 1 the pause is suddenly disrupted by M moving his shoulders up and down in small rhythmical movements while making hearable outbreaths surrounding and interwoven in the articulation of “no(h)w.” These actions are immediately reflected by a change in W's behavior from sitting still and looking into the distance to a distinct *smiling-and-gazing-behavior* directed toward M. In a flash, through the movements they share emotions building inter-affectivity. It is the totality of their “interactive expressive bodily behavior” that taken together appear as “one gestalt of shared affective experience” (Böhme et al., 2014, p. 2116). Thus, the initiation of this “laughing behavior” is built into the whole-body sense making inseparable from first order languaging behavior. Furthermore, the ending of this gestalt unit of laughing in line 6–7 comes about within a similar tight coordination of actions. Suddenly M and W inter-bodily affective dynamics are replaced by a quiet position of sitting still with their heads bowed and hands in their laps (third picture). In order to understand this sudden change we need to look at the behavior of the therapist. In the end of line 5 T starts changing her posture (see small yellow circle in second picture); she gathers her arms behind her back and then, just after M's speaking turn in line 6 (while M and W are still engaged in their laughing behavior) T closes her eyes and lets her head fall onto her chest. It is an action by which T visibly withdraws from the ongoing laughing behavior while displaying concentration and introversion as opposed to the extroverted mutual laughing exhibited by M and W. It is striking how this silent, yet overt, bodily demonstration achieves a change in the dialogical system that ultimately stops the ongoing laughing.

Laughing brings forth a “sharedness” by engaging people which is exactly the reason it is also highly sensitive to actions of disengagement. This is illustrated by the impact of the silent withdrawal of the therapist form the laughing activity; it brings the laughter to an end pointing to the fact that laughing itself *requests* participation in order to be sustained within a dialogical system. Like other languaging acts laughter is profoundly other-oriented; it requires a response in the form of more laughter to be maintained. Thus, what this analysis points to is that laughing is not only tightly bound to the inter-affective sharing and exploration of joy and amusement; it is also integrated in the overall languaging behavior and therefore it can easily be restructured and “toned down” by other languaging acts.

#### Employment of second order patterns in laughing

Looking closer at the trajectory of the laughter reveals two significant “peaks” of laughing in terms of volume, intensity, duration and postural sway in line 3–4 (overlapping) as well as line 6–7 (also partial overlapping). Common for these peaks is their sequential placement right after verbal and gestural actions; i.e., they seem to function as multimodal responses to what have just been said (and done by means of gesture) suggesting that these actions are not only built into the very structure of laughing, but even contributes significantly to its development. Now let us take a closer look at these actions.

In line 2 W makes a very distinct gesture-and-posture (see first picture) exactly at the point when M says *PAUSE FOR REFELCTI::ON:* ☺thereby providing a visual feedback and image reflecting the wordings. Likewise, in line 6 a similar (albeit not identical) gesture-and-posture is performed by M simultaneously with his own speech on *an amazi(h)ng PRESSURE*☺. We can call these repeated gesture-and-postures, an emblematic thinking-gesture-and-posture. They have the characteristics of placing the right hand or fingers either on one's cheek (first instance—see picture) or in front of the mouth (second instance) while wrinkling brows and looking downwards (somewhat like the famous “The thinker” sculpture by Auguste Rodin). These gestural actions arise from and are integrated into the whole gestalt unit of laughing in which they have a complementary function to the ongoing speech. Both of them complement the meaning of the verbal actions of having to think hard whilst under pressure; i.e., they provide an image of “concentration” that in turn can be mutually elaborated adding to the sharing of affective experiences, and thus again contributes to the humorous effect which can be witnessed by the subsequent increase in laughter following them.

Thus, we can see how the first order activities of shared laughing are constrained and enriched by second *order patterns*. The utterances themselves are at the same time first order embodied actions (smiley voice, high volume, laugh particles within the wordings, postural sway, etc.) and second order manifestations of affording a view from the outside—e.g., “here we are, a couple in therapy without even being able to (immediately) come up with something nice to say about each other.” It illustrates how languaging activity can be seen as multi-scalar, since it involves a coupling with other timescales transcending the here-and-now of situational activities. This dimension concern the second order patterns that originate from larger scale dynamics of interacting agents on larger (and longer) socio-cultural time scales. In dialogical terms it enacts “other voices” (Linell, 2009), i.e., in human interaction we do not just interact with each other, but also with an array of third parties emanating from cultural traditions, societal norms and so forth. As famously pointed out by Bakhtin: “The word in language is half someone else's. It becomes one's “own” only when the speaker populates it with his own intentions, his own accent, when he appropriates the word” (Bakhtin, 1982, p. 294). Thus, sense-making and meaning in interaction cannot be reduced to individual activity; it is, at once, inter-bodily, interactional, situated, and situation transcendent, and in that sense fundamentally *co-authored* (Linell, 2009; Steffensen, 2012, see also Cuffari, 2014, this volume):

Sense making re-enacts multiple voices, defined as *silent others* that affect what we think, say, do and not do in situated dialogue. Sense-making, thus, unfolds as *double dialogicality* that links socio-cultural history (norms, knowledge, rules etc.) with real-time dynamics as we orient toward each other and use cultural artefacts (including verbal patterns) (Pedersen and Linell, in press).

The verbal and gestural actions in line 2 and 6 “comment” on the situation by evoking a position viewing and evaluating this specific couple therapy interaction in the here-and-now from a larger “outside.” The wordings, gesture and posture invite such an outside view of socio-cultural norms that creates a doubleness (Jensen and Cuffari, 2014) that actually seems to furnish and elaborate on the humorous effect. The second order view from the outside may add to a feeling of absurdity, which, in this case, makes the situation even funnier—and in paradoxical way contributes to the inter-affective sharedness of laughing together. In this way, having a closer look at the trajectory of the laughter illustrates how “laughing” is a rich and complex affective phenomenon deriving from first order activities while being constrained by second order patterns.

#### Summary

To sum up, the affective quality of laughter as an integral part of languaging can be summarized in the following way:

- Laughter occurs as a whole-body phenomenon involving not only in- and outbreaths, but posture, facial movements, gesture, intonation, volume of speech. and tactility as well.
- Laughter is intertwined with wordings while also being deeply embedded in the inter-bodily dynamics working as a whole behavioral gestalt unit of inter-affectivity in which affect and emotion must be understood as constituting parts.
- Laughter is tightly coordinated with various communicative motives as part of a whole-body sense making which places laughter as an integral part of languaging behavior.
- Laughter as an activity can be tightly constrained second order socio-cultural patterns which can enrich and elaborate on the laughing activity.

## Final remarks

This article offers a re-specification of the traditional distinction between “language system” and “language use” as first order languaging and second order language. It is re-conceptualization that in turn offers an opportunity to see affect and emotion as part and parcel of languaging behavior while also being constrained by second order language. In that sense emotion and affect need not be separated from language; emotion and affect need not be treated as “non-linguistic elements” that are added to language. Instead languaging behavior is promoted as inherently affective and at the same time enmeshed in second order patterns. An obvious advantage of such an approach is that language can be studied as part of human action as such which again allows us to see aspects of that action, hitherto separated from language, such as affect and emotion, as part and parcel of language as it evolves from *human life* (Cowley, 2011a; Steffensen, 2014); not just as an “instrument” that we use for “communication.” This entails that language is not first and foremost seen as a system, it is not just about words, and it is not conceived of as a channel that transfers information; nor is language understood as merely a social phenomenon devoid of a biological dimension. On the contrary it is grounded in a *naturalistic* approach to language that sees language as evolved from and completely intertwined with the complexity of *human behavior*.

However, this approach to language also raises serious conceptual and methodological challenges. One of them being: if language is re-specified as whole-body sense making, or behavior, how can we, as researchers interested in language, specify, delimit and measure our object of study? Or put simply, where does languaging begin and where does it end? In a recent review article Sune V. Steffensen discusses this problem arguing that Thibault's broad definition of languaging is indeed too broad:

While first order linguistic interaction and coordination is indeed a whole-bodied achievement, the definition may seem too broad, as it can be read as suggesting that each and any “whole-bodied achievement” is an instance of first-order languaging. But describing my boiling an egg or preparing an omelette as first-order languaging intuitively seems to stretch the term. On the other hand, Thibault's definition would be applicable if wordings played a part in recalling my mother's instructions of how to make an omelette, or if I elicit my family's preferences for hard-boiled or soft-boiled eggs (Steffensen, 2014).

It is true that preparing an omelet does not intuitively seem to be part of languaging. We need to be able to able to discriminate between languaging behavior and other types of behavior. As suggested in the introduction one way to define languaging behavior more precisely is to see it as *coordinated actions constrained by second order patterns*. Such an approach is also implied in the quote above by suggestion the inclusion of wordings in recalling a recipe as a possible way of viewing cooking as an instance of languaging.

Still, such a tentative definition does not solve all the problems in conceptualizing languaging as whole body behavior or sense making. First of all, it does not sufficiently address the question of intentionality and meaning. Many types of behavior are carried out without any intention of influencing the behavior or experiences of others, but for practical purposes: We make an omelet or prepare dinner for our family in order to get something to eat; not because we want to “convey a message” (even though that might sometimes be the case). Clearly, such an activity would not count as languaging behavior; on the other hand we might imagine a very distinct way of preparing a meal, a clattering of the crockery and cutlery, i.e., a hectic, hasty, and perhaps even angry way of cooking that (granted the presence of others) may indeed be orchestrated in a way such as to cause “an experience that happens to coincide with the narrow situation or the larger reality such as it is enacted, and has to be mapped against the environmental medium, including the psychological environment” (Bottineau, 2010, p. 278). Even if such a behavior is performed without the use of words it might still be (partial) communicative deliberate by its virtue of doing, acting and manipulating the environment in certain ways which transcends the mere practical purposes. Furthermore, “making an omelet” or “preparing a meal for a family dinner” are practices that are only possible within a specific ecological niche with certain historic-social-cultural horizons of significance, i.e., it is by no means detached from second order patterns. Likewise, cooking activities often require a certain culture-specific training; they often have a social character and perhaps even an emotional significance for the people involved. Does it count as first order languaging behavior then? There is no easy answer to this, and many further studies need to be performed in order to investigate further how languaging are enmeshed in human practices.

This article presents an ecological approach to language and emotion. One of the implications of such a point of departure is that the distinctions between what is considered biological vs. social are fundamentally challenged. Is preparing a meal a social or biological act? Or for that matter engaging in learning activities with a speech and language therapist or participating in couple therapy with your spouse? From the direction of this work, posing these questions makes little, if any, sense. This article argues for a reconsideration of the unreflective rift between the biological (individual) and the social (collective). Mainstream linguistics and cognitive science generally take biology as first and foremost an individual phenomenon, while sociality is understood as something purely collective and public. Correspondingly, emotion and cognition are construed as individual, internal, and private processes, while communication conversely is conceived as purely social, public, and outer. The problem arises when these distinctions come off as mutually exclusive. On a dichotomous reading, what is social is understood as that which by definition does not belong to nature or biology and the other way round (Cuffari and Jensen, 2014). However, the notion of ecology rests on a principal bio-social foundation; unlike the more familiar, and wholly social, concept of context:

The ecology is not an outer frame that just surrounds or contains the individual agents and it cannot be captured in the simple outer-inner dichotomy. Rather, the ecology emerges from the active sense-making of agents employing the physical materials and socio-cultural resources of the environment (Cuffari and Jensen, 2014).

In the same vein, we need to transcend the dichotomy between viewing emotions as either a primarily biological or social phenomena. Emotions are at the same time rooted in neurological structures and embodied sensations, subjectively felt experiences, socially embedded and integrated with action and languaging. In that sense, emotions are part and parcel of our ecology in the manner of which they are intertwined with our languaging behavior in the animal(human)-environment system. Embodied emotional actions are enacted in languaging as affordance to locate and orient us to the possibilities that we encounter. In that sense, emotions help us to build an interpersonal “geography” for us to share, participate in or confront.

### Conflict of interest statement

The author declares that the research was conducted in the absence of any commercial or financial relationships that could be construed as a potential conflict of interest.

## Footnotes

^1^Hence, when referring to languaging as behavior or whole-body sense making it is in the Thibault version of the term. For an overview of the various positions to languaging and the first and second order distinction, see Steffensen (2014).

^2^The specific focus on emotion in situ, in the observable here and now of social interaction, entails that what is often referred to as “autobiographical” emotional experience (Damasio, 2003), that is, emotional memories and knowledge about the past, will, for the most part, play a less prominent role in the following. Whereas what is commonly called “procedural” emotional experience (Tulving, 1984), or *emotional episodes* (Colombetti, 2014), that is, momentarily emotional experiences and action embodied in person's behavior, play a much more prominent role in the analyses as well as the theoretical chapters.

^3^Some scholars distinguish between affect (or mood), as a more primary and pervasive phenomenon, and emotion, as more experientially specific and distinct (see for instance Colombetti, 2014, pp. 2–15). However, for the sake of simplicity and space this discussion will not be pursued in the present work. Thus the terms “emotion” and “affect” are used more or less interchangeably also reflecting the various uses within linguistics and psychology respectively. “Affect” is a more common term in linguistics whereas “emotion” is more widespread in the social sciences and psychology. For this reason both terms are used here but with “emotion” as the most prevalent one.

^4^An alternative to view emotion and affect as inherently evaluative can be found in recent enactive approaches to emotion: “From the enactive standpoint defended here, bodily arousal is not merely a response to the subject's evaluation of the situation in which he or she is embedded. It is rather the whole situated organism that subsumes the subject's capacity to make sense of his or her world” (Colombetti, 2010, p. 157).

^5^See for instance this introductory line presenting the study of pragmatics: “Those aspects of language use that are crucial to an understanding of language as a system, and especially to an understanding of meaning, are the acknowledged concern of linguistic pragmatics” (Levinson, 1983, Back cover).

^6^The claim is that with the invention of writing the notion of language underwent a process of reification and objectification due to the permanent and visible signs on paper. The conception of language was transformed from an embodied activity into an object (of study). The view on language as a structured set of abstract forms used to represent things in the world evolves from this written dimension and its embodied dialogical nature is backgrounded or treated as irrelevant (Linell, 2005).

^7^This is mostly true of communication studies whereas as other fields, such as gesture studies, to a much larger degree see verbal utterances and gesture as one communicative whole and therefore gesture as a part of language (Kendon, 2004). Still, surprisingly only recently have gesture (arm and hand movements) been directly related to emotion and affect. More about this in section Languaging, primary intersubjectivity, and language.

^8^It is of course important to mention that the information transfer model has been heavily criticized for exactly this and is now abandoned by many positions within the social sciences. The more up to date alternative is a view on meaning in interpersonal communication as a co-constructed sense-making that is accomplished within the interaction itself (Jensen, 2014).

^9^Today there is to large extent an agreement within emotion researchers on the validity on cross cultural facial expressions and their correspondence to basic emotions. At least when it comes to the universality of distinguishing between negative emotions such as happiness, sadness, fear, anxiety on the one hand and positive emotions as surprise and joy on the other hand. It is for instance generally acknowledged that it is a universal phenomenon that eyes widen with surprise and joy and narrow with anger. Still, it has proven more difficult to distinguish within different negative emotional expressions—such as sadness, anger, fear, and disgust—than between positive emotions such as joy and happiness (Planalp, 1998).

^10^Evidently, this problem also concerns the distinction between verbal and nonverbal communication: “A long standing debate concerning verbal and nonverbal communication has been whether verbal communication can be trusted at all in terms of its ‘truthfulness.’ In almost any introductory textbook to nonverbal communication, students learn that words may lie, and nonverbal signals do not” (Sandlund, 2004, p. 84).

^11^See Goodwin et al. (2012) for a more broad approach to stance including bodily behavior as well.

^12^For other ways of capturing and transcribing languaging behavior see Steffensen (2012), Pedersen and Steffensen (in press), and Böhme et al. (2014).

^13^This recording was made available to me by professor, Gitte Rasmussen, on condition that the anonymity of all the persons involved was upheld. I would like to express my deepest gratitude to Gitte Rasmussen for the opportunity to work on these data.

^14^At the same time however, for a brief moment, this structural-emotional-coupling isolates E as not being part of this alignment, which is apparent in the way he looks down at his own cards disengaging from the shared activities between S and M.

^15^For instance, the *emotional contagion* approach explicates how emotional reactions such as laughter rapidly spread in groups of both mammals and humans. It is a process consisting of three steps—mimicry, feedback, and contagion—enabling people “from moment-to-moment to catch others' emotions” (Hatfield et al., 1993, p. 99).

^16^The recordings were undertaken in relation to my Ph.d. dissertation in 2008 in collaboration with the Danish Imago Center.

^17^As investigated by Gail Jefferson laughter in interaction sometimes has the social effect of dealing with sensitive topics. It can be seen as a way of managing troubles-talk “exhibiting that, although there is this trouble, it is not getting the better of him [the speaker]; he is managing; he is in good spirits and in a position to take the trouble lightly (Jefferson, 1984:, p. 351). Something similar seems to be the case here. By engaging in laughing behavior the couple mutually deals with the fact that they were not, on the spot, capable of recalling something valuable about each other.
